# Lower knowledge about diabetes among foreign‐born compared to Swedish‐born persons with diabetes—A descriptive study

**DOI:** 10.1002/nop2.217

**Published:** 2018-11-22

**Authors:** Sara Pettersson, Emina Hadziabdic, Helén Marklund, Katarina Hjelm

**Affiliations:** ^1^ Department of Social and Welfare Studies Linköping University Norrkoping Sweden; ^2^ Department of Health and Caring Sciences, Faculty of Health and Life Sciences Linnaeus University Vaxjo Sweden; ^3^ Department of Public Health and Caring Sciences Uppsala University Uppsala Sweden

**Keywords:** diabetes mellitus, knowledge, migrants, nursing, refugees

## Abstract

**Aim:**

To compare foreign‐ and Swedish‐born persons, diagnosed with type 2 diabetes, to study whether there are dissimilarities in knowledge about diabetes and to study determinants of knowledge.

**Design:**

A cross‐sectional descriptive study was conducted.

**Method:**

Data were collected between September 2014 and March 2016, using the standardized Diabetes Knowledge Test (DKT), statistically analysed.

**Results:**

The results showed dissimilarities in knowledge between foreign‐ and Swedish‐born persons, supporting the hypothesis that foreign‐born persons had lower knowledge about diabetes than Swedish‐born persons. There was a relationship between poor knowledge and country of birth, marital status and employment status. Country of birth was the strongest independent determinant of knowledge about diabetes. The risk of poor knowledge was ten times higher among persons born in the Middle East or in another country outside Europe compared with Swedish‐born persons. Other influencing factors for poor knowledge about diabetes were being not gainfully employed and living alone.

## 1 INTRODUCTION

Type 2 diabetes constitutes a global public health problem which is rapidly increasing and particularly affecting vulnerable populations in society, such as migrants (Guariguata et al., 2014; IDF, 2015; WHO, 2015). Migrants include foreign‐born persons who have moved to another country voluntary as immigrants or forced as refugees (IOM, 2017). It has been found that knowledge is important to be able to perform necessary self‐care to promote health and prevent complications related to diabetes; thus, there is a need for studies that contribute knowledge about migrants and what they know about the disease (Testa, Bonfigli, Genovese, & Ceriello, 2015). One of the nurse’s main tasks is to support patients in self‐care by teaching, and thus, assessment of knowledge about the disease is important (Leininger & McFarland, 2006). However, no previous studies have been found examining knowledge about diabetes among foreign‐born persons compared with a native population in a host country.

## 2 BACKGROUND

Today approximately 422 million persons are affected with diabetes mellitus (DM), and the prevalence is predicted to reach 592 million in 2035 (Guariguata et al., 2014); about 85% have type 2 diabetes (IDF, 2015, WHO, 2015). Type 2 diabetes is a progressive disease with the risk of developing micro‐ and macrovascular complications, influencing health negatively and possibly leading to high costs for health care and suffering for the individual (IDF, 2015, Socialstyrelsen, 2015). Although serious complications may develop, the disease can be controlled, complications can be prevented and the incidence can be delayed by practicing adequate self‐care. For patients with type 2 diabetes, active participation in self‐care based on knowledge about the disease is thus of great importance (IDF, 2015).

The risk of being affected with type 2 diabetes is increased in migrants, particularly those from countries outside Europe, compared with the native population of the host country and this is an increasing problem for several European countries (IDF, 2015; Shaw, Sicree, & Zimmet, 2010; Whiting, Guariguata, Weil, & Shaw, 2011; Zimmet & Alberti, 2006). This leads to personal suffering as well as public health challenges and high costs (IDF, 2015). Sweden, like many other European countries, has changed to a multicultural society due to ongoing global migration (SOS, 2015, SCB, 2016). Today, about 17% of the population in Sweden are born abroad (SCB, 2016). The migrant population includes more than 200 different nationalities, with the largest groups coming to work voluntarily as labour migrants from Finland and former Yugoslavia and during the 1990s also as refugees forced to flee from the war (SOS, 2015, SCB, 2016). During the last decade, migration to Sweden has been dominated by persons of non‐European origin, with the largest group being refugees from the Middle East. Most non‐European migrants coming to Sweden are refugees.

Health‐related behaviour, including self‐care, is determined by beliefs about health and illness, held by the individual and culturally determined, based on the person’s knowledge and refined by experiences (Glanz, Rimer, & Lewis, 2008; Hjelm & Bard, 2013; Hjelm, Bard, Nyberg, & Apelqvist, 2003, 2005). The behaviour is also influenced by whether the person perceives the causes of health and disease as possible to influence or not, in other words having an internal vs. an external locus of control (Rotter, 1966). Furthermore, behaviour is influenced by the perceived risk, threat or susceptibility of being affected by a disease, the perceived seriousness of the disease and the likelihood of action due to perceived benefits or barriers/costs to behaviour change required by the disease (Rosenstock, Strecher, & Becker, 1988). Other influencing factors are, for example age, gender, education, ethnic origin and experiences related to migration Rosenstock et al. (1988) and Glanz et al. (2008). As regards type 2 diabetes, self‐care is crucial for disease control and prevention of diabetes‐related complications (IDF, 2015). A person should receive individualized information about their health and health‐related behaviour, and the information should be individually tailored and culturally appropriate (Leininger & McFarland, 2006). Thus, it is important to meet the needs of people from different countries and healthcare staff must have an understanding and knowledge of how cultural background affects perceptions of health and illness (Leininger & McFarland, 2006). Culturally adapted or appropriate care or health education can be defined as education tailored to the cultural and religious beliefs and linguistic skills of the community being approached, but also taking into consideration likely literacy skills (Hawthorne, Robles, Cannings‐John, & Edwards, 2008). In Sweden, persons diagnosed with type 2 diabetes are generally managed in primary healthcare centres or in specialized in‐hospital‐based clinics in the case of complications related to diabetes. Irrespective of origin, all patients should receive the same care (SFS, 2017). The exception is that non‐Swedish‐speaking persons, according to the law, have the right to get an interpreter in all contacts with authorities (SFS, 1986). However, access to interpreters varies and can be limited, and therefore, many consultations in health care are made without an interpreter or using relatives (Hadziabdic & Hjelm, 2014).

In the literature review, no previous studies comparing knowledge about diabetes between foreign and a native population have been found. A study that indicates limited knowledge about diabetes among Turkish migrants diagnosed with diabetes, living in Germany (Kofahl, Knesebeck, Hollman, & Mnich, 2013), has been found. Further, a few studies support this path, aiming to implement and evaluate intervention programmes that measure the knowledge about diabetes in different migrant groups of Korean (Choi & Rush, 2012; Song, Han, & Lee, 2010), Chinese (Chesla, Chun, & Kwan, 2013; Sun, Tsoh, Saw, Chan, & Cheng, 2012) and Bangladeshi (Islam, Wyatt, & Patel, 2013) origin living in the USA as an outcome measure.

Qualitative studies comparing foreign‐ and Swedish‐born persons with type 2 diabetes have indicated dissimilarities in knowledge and perceived seriousness of diabetes, with limited knowledge and lower risk awareness, which might affect health‐related behaviour including self‐care (Hjelm & Bard, 2013; Hjelm et al., 2003, 2005). Thus, it was hypothesized that foreign‐born persons have lower knowledge about diabetes than Swedish‐born persons. The aim of the study was to compare foreign‐ and Swedish‐born persons, diagnosed with type 2 diabetes, to study whether there are dissimilarities in knowledge about diabetes mellitus and to study determinants of knowledge.

## 3 THE STUDY

### 3.1 Design and method

This was a cross‐sectional descriptive study with data collected with a standardized and validated self‐report instrument, Diabetes Knowledge Test (DKT; Fitzgerald et al., 1998), to investigate knowledge about diabetes mellitus. The design was chosen to give the possibility to gather information about the variable knowledge in migrants with type 2 diabetes and to enable studies of relationships with the person’s background characteristics (Creswell, 2014).

### 3.2 Participants

A convenience sampling procedure was used. In this study, a distinction will be made between people born in Sweden and abroad and people born in another country than Sweden are considered as foreign‐born persons (SOS, 2015). Invited were all known foreign‐born persons diagnosed with type 2 diabetes according to ICD E11 (WHO, 2015) managed in a diabetes clinic in a healthcare centre in an immigrant‐dense area in a county in Sweden, aged ≥18 years and with duration of diabetes ≥1 year. Persons with known psychiatric diagnoses ICD F00–F29/F60–F99 (WHO, 2015) were excluded since cognitive impairment might influence the results. Inquiries, to participate in the study, were sent to persons who met the inclusion criteria (*N* = 379), with reminders 3 weeks apart. Fifty‐two persons did not want to participate in the study, 14 inquiries came back since the person could not be reached at the current address and two persons did not understand the interview questions, despite an interpreter being present and were thus excluded; 242 persons did not answer. Of the foreign‐born persons, 69 were included in the study. The subsequent process was to recruit 69 Swedish‐born persons, matched for gender, by the same procedure as the foreign‐born group. The study population did not differ from the non‐respondents as regards gender, age and country of birth (*p* = 0.49, 0.66, 0.82).

### 3.3 Data collection

Data were collected between September 2014 and March 2016 by structured interviews based on questionnaires. In this study, the Diabetes Knowledge Test (DKT; Fitzgerald et al., 1998) and socio‐demographic background data are reported. Permission for the study was received from the operation manager after oral and written information about the study from the principal investigator (PI; first author). Then, the diabetes specialist nurses at the clinic were informed, orally and in writing, about the implementation of the study. After this, one diabetes specialist nurse identified persons meeting the inclusion criteria, from computerized registers at the clinic. Then, a letter with information about the study and a prepaid response envelope, addressed to the clinic, was sent. Two reminders, with 3 weeks apart, were sent in the event of no answer. The inquiry was translated into Arabic and Bosnian/Croatian/Serbian, as they are the main language groups among migrants in Sweden (SCB, 2016) and was included in the letter to persons of this origin. The Bosnian/Croatian/Serbian and the Arabic version were both translated by authorized interpreters.

The structured interview, including all instruments, lasted about 45–60 min and was held by a registered nurse and in the presence of an authorized interpreter (*N* = 40) when so desired, in a secluded room at the diabetes clinic. Further, in connection with the interview, glycaemic control (HbA1c) was tested. HbA1c was measured with latex agglutination inhibition and total haemoglobin concentration measured with potassium. The fractional percentage of HbA1c was then calculated and expressed as mmol HbA1c per mol of Hb according to Siemens 2008.

A pilot test was performed in ten foreign‐born persons to assess how the interview guide worked. Face and content validity were tested (Polit & Beck, 2012). The instrument worked well, and therefore, all interviews were included in the study.

The Diabetes Knowledge Test (DKT), developed by the Diabetes Research and Training Center at the University of Michigan (Fitzgerald et al., 1998), was used as one of the several instruments, after approval was obtained from the copyright holder. DKT is a questionnaire that consists of 23 items, measuring a person’s knowledge about diabetes in a general part (items 1–14), an insulin use part (items 15–23) and a total part (items 1–23). For every item, there are several alternative answers. In this study, the general part was used for all participants while the insulin part, and thereby also the total part, was answered by insulin‐treated participants only (see Table 1). DKT has demonstrated adequate validity and reliability, Cronbach’s alpha > 0.70 (Fitzgerald et al., 1998) and has been adapted for use in many countries throughout the world and translated by researchers into several languages (Fitzgerald et al., 2016). DKT was translated into Swedish and back‐translated into English by two different authorized professional translators. Translation into Swedish was done in several steps to ensure that the essential meaning of the items was preserved (Polit & Beck, 2012). The original questionnaire in English was translated into Swedish by an authorized professional translator and then retranslated back into English by another authorized professional translator. The PI for the study then reviewed the two English versions and agreed that they were equivalent. The interviews were performed with professional interpreters in respective language. DKT has been used in several study populations with different origin and languages (Chesla 2012; Choi & Rush, 2012; Mufunda, Albin, & Hjelm, 2012; Islam et al., 2013; Kofahl et al., 2013).

**Table 1:** Participant distribution based on country of birth and area, presented by frequency

Country	*N*
Sweden	69
Europe	
Bosnia	9
Turkey	8
Poland	4
Finland	4
Kosovo	3
Croatia	2
Italy	1
Middle East/outside Europe	
Syria	14
Iraq	12
Chile	5
Lebanon	3
Sri Lanka	1
Burma	1
Burundi	1
Somalia	1
Total	138

### 3.4 Data analysis

To describe the data, numbers and percentage, mean (*SD*) and median (range) were used (Altman, 1994). Comparisons between groups were made by tests of statistical significance. For continuous variables, Student’s *t* test was used for normally distributed variables and Mann–Whitney *U*‐test for non‐normally distributed variables and chi‐squared test was used to ascertain any differences between categorical variables; *p* < 0.05 was considered statistically significant.

To determine diabetes knowledge measured by the DKT (Fitzgerald et al., 1998), one point was awarded for a correct answer and zero for a wrong answer or no response. The total knowledge score ranged from 0 to 23 and was categorized as: <11 = poor knowledge, 11–17 = average knowledge and >17 good knowledge (Al‐Adsani, Moussa, & Al‐Jasem, 2009). The total sum of points was calculated, and knowledge deficit arose when over 50% of the questions were incorrectly answered (Al‐Adsani et al., 2009). The part for general knowledge (questions 1–14) was categorized as follows: <7 = poor knowledge, 7–11 = average knowledge and >11 good knowledge. The part for insulin use (questions 15–23) was categorized as: <5 = poor knowledge, 5–7 = average knowledge and >7 = good knowledge.

For analytical statistics, to identify any independent associations between knowledge and socio‐demographic variables and diabetes‐related characteristics, multiple logistic regression analysis (stepwise logistic regression, backward conditional) with calculation of odds ratio (OR; 95% CI) was used (Altman, 1994). The analysis was performed in several steps with different independent variables associated with the dependent variable poor knowledge, measured in the part for general knowledge in the DKT, 0–6 points (Al‐Adsani et al., 2009). Variables with *p* < 0.1 in bivariate analysis were chosen as covariates and entered as categorical variables (see Table 5). Country of birth, marital status, employment status and educational level turned out to be significant variables. Country of birth included three indicators: Born in Sweden, European countries or Middle East/countries outside Europe. Marital status was dichotomized into living together (being married or cohabitant) or alone (unmarried, divorced or widow/er). The variable employment status was dichotomized into gainfully employed (gainfully employed or student) or not (unemployed, on sick leave, or retired). Educational level was divided into low (no/primary/secondary education) or high (university < 2 > years). Hosmer and Lemeshow (1980) chi‐squared test of goodness of fit was used to assess how well the model fits with a significance level of 0.05 (5%). Calculations were made in Statistical Package for the Social Sciences (SPSS, version 23).

### 3.5 Ethics

The study was approved by a Regional Ethics Committee and implemented in accordance with the Helsinki Declaration (World Medical Association, 2013). Written informed consent was obtained from the respondents. All collected data were anonymized and coded so that no participant could be identified. Collected data were stored in a locked space at the PI’s workplace and inaccessible to the healthcare staff. Results were analysed and presented on a group level and in such a way that no one could be identified.

## 4 RESULTS

### 4.1 Respondents’ socio‐demographic and diabetes‐related data

The study population included 138 participants, both men and women, of whom 69 were foreign‐born persons, aged 33–90 years and 69 were Swedish‐born, aged 48–91 years. The foreign‐born group included mainly persons born in the Middle East and former Yugoslavia (Table 1).

In the foreign‐born group, the majority (75%) had received their diabetes diagnosis in Sweden (Table 2). Most were refugees (73%) and had been residents in Sweden for a mean 25 years, varying from 3 to 44 years. The foreign‐born compared with the Swedish‐born group was younger (*p* = 0.001), had a lower level of education, (*p* = 0.010), shorter duration of type 2 diabetes (*p* = 0.041) and poorer glycaemic control (*p* = 0.001).

**Table 2:** Socio‐demographic and diabetes‐related characteristics of respondents

Variable	Swedish‐born *N* (%)	Foreign‐born *N* (%)	*p*‐Value
Gender			
Male	37 (54)	37 (54)	
Female	32 (46)	32 (46)	
Age (years)			
18–64	25 (36)	44 (64)	0.001
≥65	44 (64)	25 (36)	
Marital status			
Unmarried	11 (16)	2 (3)	0.057
Married	37 (54)	49 (71)	
Cohabitant	6 (9)	4 (6)	
Divorced/separated	5 (7)	7 (10)	
Widow/widower	10 (14)	7 (10)	
Educational level			
No education	0 (0)	10 (15)	0.010
Primary school	25 (36)	27 (39)	
Secondary school	24 (35)	17 (25)	
University ≤ 2 years	7 (10)	9 (13)	
University > 2 years	13 (19)	6 (9)	
Duration of Diabetes in years			
<10	28 (40)	39 (58)	0.041
≥10	41 (60)	30 (42)	
Employment status			
Gainfully employed	20 (29)	20 (29)	0.001
Unemployed	2 (3)	9 (13)	
Student	0 (0)	2 (3)	
Sick leave	0 (0)	9 (13)	
Early retirement	10 (14)	11 (16)	
Retired	37 (54)	18 (26)	
Diabetes treatment regimen			
Diet	9 (13)	10 (15)	0.143
Oral agents	33 (48)	38 (55)	
Insulin	16 (23)	8 (12)	
Combination of oral agents and insulin	11 (16)	13 (19)	
Glycaemic control (mmol/mol)			
Good < 52	26 (38)	35 (51)	0.001
Acceptable 52–69	40 (58)	21 (30)	
Poor ≥70	3 (4)	13 (19)	
Cause of migration			
Refugee		50 (73)	
Labour		7 (10)	
Relative		12 (17)	
Years of residence in Sweden, mean (*SD*)		25 (17)	

### 4.2 Diabetes knowledge

The Swedish‐born group had significantly higher knowledge in all three parts of the instrument, compared with the foreign‐born group (Table 3). In the general part, the mean value was 66 (*SD* 21) vs. 46 (*SD* 26); in the insulin part, it was 57 (*SD* 22) vs. 17 (*SD* 33); in the total part, it was 64 (*SD* 20) vs. 31 (*SD* 32).

**Table 3:** Knowledge level about diabetes, Swedish‐ and foreign‐born persons, diagnosed with type 2 diabetes, measured by the Diabetes Knowledge Test (DKT)

Variable	Swedish‐born Mean (*SD*)	Foreign‐born Mean (*SD*)	*p*
General knowledge	66 (21)	46 (26)	<0.001
Insulin use knowledge	57 (22)	17 (33)	0.003
Total knowledge	64 (20)	31 (32)	0.008

A comparison of intervals of general knowledge level about diabetes between foreign‐ and Swedish‐born persons with type 2 diabetes is presented in Table 4. The number of persons having poor knowledge about DM (<7 points) was higher in the foreign‐born group (36 vs. 14) in contrast to a higher number with good knowledge (>11 points) among Swedes (18 vs. 2).

**Table 4:** Respondents’ knowledge about diabetes measured by the Diabetes Knowledge Test (DKT)

General knowledge	Swedish‐born (*N*)	Foreign‐born (*N*)	Description
<7	14	36	Poor
7–11	37	31	Average
>11	18	2	Good

### 4.3 Determinants of knowledge about diabetes

The multifactorial influence on knowledge (Table 5), the general part, showed that the most important determinant for poor knowledge was country of birth. The risk of poor knowledge was almost ten times higher among persons born in the Middle East or in another country outside Europe (OR = 9.7; 95% CI 3.2–29) and five times higher among persons born in a European country (OR = 5.0; 95% CI 1.9–13.7) compared with Swedish‐born persons. Other influencing factors were not being gainfully employed (OR = 5.5; 95% CI 1.9–16.4) and marital status in terms of living alone (OR = 2.9; 95% CI 1.8–7.1).

**Table 5:** Multifactorial influence on knowledge about diabetes, in persons diagnosed with type 2 diabetes. Results of multiple regression analysis with significant influence on poor level of knowledge, shown for independent variables

Dependent variable	Independent variable a	Regression coefficient b	*SEM*	Odds ratio (CI 95%)	*p*‐Value
Poor level of knowledge: <7 score	Country of birth				
	Middle East/outside Europe	2.270	0.560	9.7 (3.2–29.0)	0.000
	Europe	1.611	0.512	5.0 (1.9–13.7)	0.002
	Employment status				
	Not gainfully employed	1.712	0.554	5.5 (1.9–16.4)	0.002
	Marital status				
	Living alone	1.058	0.459	2.9 (1.8–7.1)	0.021

Note

Multiple regression analysis (stepwise logistic regression, backward conditional) was performed in SPSS. Variables with *p* < 0.1 in bivariate analysis were chosen as covariates and entered as categorical variables. Country of birth included three indicators: Born in Sweden, European countries or Middle East/countries outside Europe. Marital status was dichotomized into living together (being married or cohabitant) or alone (unmarried, divorced or widow/er). The variable employment status was dichotomized into gainfully employed (gainfully employed or student) or not (unemployed, or sick leave or early retirement or retired). Besides the independent variables shown, the variable educational level, which was divided into low (no/primary/secondary education) or high (university <2 > years) was included in the model as factors that affect knowledge about diabetes mellitus. Hosmer & Lemeshow was calculated, *p*‐value at 0.517.

### 4.4 Knowledge gaps

Knowledge deficit, defined as over 50% incorrect or missing answers, was noted in the general part in eight questions in the foreign‐born group, compared with four questions in the Swedish‐born group (Table 6). Knowledge deficit in the foreign‐born group was, in descending order, related to the definition of a “free food” (85.5%), the purpose of testing HbA1c (72.5%), food that should not be used to treat low blood glucose (69.6%), what numbness and tingling may be symptoms of (68.1%), which food is highest in fat (56.5%) or in carbohydrates (55.1%) and which problem is usually not associated with diabetes‐related complications (53.6). Among the Swedish‐born persons, knowledge deficit was noted in four questions, in descending order: Which food is highest in fat (72.5%), definition of a “free food” (60.9%), what numbness and tingling may be symptoms of (58%) and food that should not be used to treat low blood glucose (55.1%).

**Table 6:** Knowledge deficit in eight questions for the foreign‐born group and in four questions for the Swedish‐born group. Correct answer is italicized

Question + Answer alternatives	Incorrect answer (%)	Incorrect answer (%)
	Swedish‐born	Foreign‐born
Which of the following is highest in fat?		
*Low fat milk*	73	57
Orange juice		
Corn		
Honey		
Which of the following is a free food?		
Any unsweetened food Any dietetic food	61	86
Any food that says “sugar free” on the label		
*Any food that has <20 calories per serving*		
Glycosylated haemoglobin (Haemoglobin A1) is a test that is a measure of your average blood glucose level for the past:		
Day	39	73
Week		
*6–* *10 * *weeks*		
6 months		
Which should *not* be used to treat low blood glucose? 3 hard candies		
½ cup orange juice	55	70
*1 cup diet soft drink*		
1 cup skim milk		
Numbness and tingling may be symptoms of: Kidney disease		
*Nerve disease*	58	68
Eye disease		
Liver disease		
Which of the following is usually *not* associated with diabetes?		
Vision problems	28	54
Kidney problems		
Nerve problems		
*Lung problems*		

## 5 DISCUSSION

This study is unique since there are no studies investigating data about migrants and their knowledge about diabetes, compared with a native population in a host country. The result of this study confirmed the hypothesis that foreign‐born persons had lower knowledge about diabetes, compared with Swedish‐born persons, as indicated in previous qualitative studies (Hjelm & Bard, 2013; Hjelm et al., 2003, 2005 ). Country of birth was the strongest independent determinant of knowledge about diabetes. The risk of poor knowledge was ten times higher among persons born in the Middle East or in another country outside Europe and five times higher among persons born in a European country compared with Swedish‐born persons. Other influencing factors for poor knowledge about diabetes were as follows: being not gainfully employed and living alone. However, as there are no previous studies comparing knowledge about diabetes among foreign‐born persons and the native population of the host country, comparisons can only be made partly. In groups consisting solely of migrants, originating from Korea (Choi & Rush, 2012; Song et al., 2010), China (Chesla et al., 2013; Sun et al., 2012) and Bangladesh (Islam et al., 2013), limited knowledge has been shown, as a result of interventions aiming to improve knowledge about diabetes in migrants. These studies show that the migrants’ knowledge initially was limited, which needs to be considered in diabetes care and particularly when designing management strategies, including teaching activities as recommended (Creamer, Attridge, Ramsden, Cannings‐John, & Hawthorne, 2015; Hawthorne et al., 2008; Socialstyrelsen, 2015). This study showed lack of knowledge in the areas of diet, how to achieve glycaemic control and dealing with diabetes‐related complications among the foreign‐born group, and therefore, future patient education for this group should focus on these areas of knowledge.

In this study, the majority of foreign‐born persons (75%) had received their diagnosis in Sweden and thus ought to have had the same diabetes education as Swedes (Socialstyrelsen, 2015). Studies have shown a lack of culturally adapted diabetes education for persons of foreign background living in Sweden (Socialstyrelsen, 2015) and in other countries (Creamer et al., 2015; Hawthorne et al., 2008), which needs to be considered.

However, the main results showed a difference in knowledge between the foreign‐born and the Swedish‐born group and to be able to improve knowledge about diabetes among foreign‐born persons, possible reasons for the difference need to be identified. To start with, it is prerequisite that the person understands information given if a person’s knowledge about an illness is to be improved. Hadziabdic and Hjelm (2014) highlighted the use of a professional interpreter during patient visits, to avoid communication barriers and to improve the impact on quality of health care, but it was not always obvious due to lack of authorized professional interpreters. The next step will be to understand which determinants knowledge is based on. According to Rosenstock et al. (1988) and Glanz (2008), health‐related behaviour, including self‐care, is determined by beliefs about health and illness held by the individual, based on the person’s knowledge and refined by experiences. Therefore, it is essential to identify a person’s health beliefs and attitude towards an illness to improve knowledge and to know whether a person perceives a disease as a threat or not (Hjelm et al., 2005). The feeling of not being able to influence a situation, in other words having an external locus of control, might also affect the acquisition of knowledge by leading to less interest in learning more about an illness (Rotter, 1966).

Further, limited knowledge about a disease might result in low‐risk awareness and perceived low severity of health and illness and low basic knowledge about the body, diabetes and health might affect how a person can understand information given (Hjelm et al., 2003, 2005 ).

Previous research has emphasized the importance of knowing a person’s migratory background, which might affect the acculturation process including self‐care behaviour and knowledge ingestion (Berry, 2005; Hjelm & Bard, 2013; Hjelm et al., 2003; Hull, 1979). Today, the migrant population in Sweden has expanded to include migrants from countries outside Europe as well as European countries. Due to push‐factors such as war and political conflicts, a large share of migrants are coming to Europe from the Middle East, Africa and Asia as refugees (SCB, 2016). In this study, 73% of the foreign‐born group stated that they were refugees and 84% of those were born in the Middle East or in another country outside Europe. Thus, it is important to consider the impact of migration background in terms of being a refugee, since many have been exposed to traumatic experiences such as war and conflicts and previous research has indicated that persons fleeing from traumatic situations might suffer from post‐traumatic stress disorder (PTSD; Shawyer, Enticott, Block, Cheng, & Meadows, 2017; Sundquist, Bayard‐Burfield, Johansson, & Johansson, 2000), which has been shown to act as a barrier to receiving information (Hjelm et al., 2005).

When considering migratory background and origin in the studied population, mainly refugees from the Middle East and former Yugoslavia, the cultural distance compared with the Swedish population was large, for example in language, social relations, family structure and cultural values (Triandis, 2000). Thus, it can be difficult to understand each other in a patient education situation and particularly as there is a lack of culturally appropriate and culturally adapted education models in diabetes care (Creamer et al., 2015; Hawthorne et al., 2008; Socialstyrelsen, 2015). Thus, it is important to consider that persons coming from countries with great cultural distance might experience difficulties in teaching situations (Triandis, 2000). Research has demonstrated that when migration was self‐initiated, the acculturation process, including adaptation to a new situation, for example teaching situations, became more positive (Hull, 1979), and thus, the acculturation stress decreased (Berry, 2005). Acculturation stress which might also be another barrier against information delivered.

In addition to the migration situation that might act in different ways as a barrier to adopting new knowledge, most diabetes education is delivered when the person is newly diagnosed. This is often at the same time as the patient is in a crisis reaction, due to having been informed about having a chronic diagnosis (Cullberg, 2006). This might also be a barrier to information, as we know that during the crisis reaction many are mentally blocked, and this may cause difficulties in assimilating new knowledge. The question is what repeated information and teaching there has been for this particular group over time. Another influencing factor is the shorter duration of diabetes in foreign‐born compared with Swedish‐born persons, and thus, they have had less contact with health care and exposure to diabetes‐related knowledge.

Research has shown that a good social network can have a positive effect on self‐care behaviour such as knowledge ingestion (Berterö & Hjelm, 2010). Being unemployed and/or living alone could lead to limited social network, which previous studies have emphasized as playing an important role in supporting positive self‐care behaviour among persons with diabetes (Lanting et al., 2008), and hence, the absence of a social network can affect the way a person manages their disease and gathers knowledge about it (Berterö & Hjelm, 2010). In this study, foreign‐born compared with Swedish‐born persons were to greater extent unemployed and on sick leave and had a higher risk of poor knowledge about DM than those who were gainfully employed. In addition, the study showed that participants living alone had a higher risk of poor knowledge than those living with a partner. Migrants in Sweden, particularly those of non‐European origin, are more often unemployed and living in limited social networks (Socialstyrelsen, 2009).

A person’s self‐care ability is related to individual beliefs about health and illness, which form their attitudes to self‐care and which are culturally determined and based on their knowledge, and thus, it is of great importance to assess these and base all educational activities on them to achieve a successful acculturation process and increase knowledge about an illness and improve health (Leininger & McFarland, 2006). Moreover, according to the Swedish Health and Medical Services Act, a person should be given individualized information about their health and health‐related behaviour (SFS, 2014, 2017 ).

The results of this study showed that foreign‐born persons living in Sweden, born in the Middle East or in another country outside Europe, who were living alone or were not gainfully employed were liable to have poor knowledge about diabetes.

### 5.1 Study limitations

The sample size might be seen as limited, but analysis of dropouts did not show any significant dissimilarities compared with the listed population of foreign‐born persons diagnosed with type 2 diabetes concerning gender, age and country of birth. However, the study population is representative of how the migrant population in Sweden is composed, mainly with persons from the Middle East and former Yugoslavia (SCB, 2016).

## 6 CONCLUSION

Foreign‐born persons with type 2 diabetes, particularly those from a country outside Europe, living in Sweden, had lower knowledge about diabetes compared with Swedish‐born persons.

For future health policy and services, it is of great importance to assess person’s own knowledge and develop appropriate individualized education including diet, glycaemic control and diabetes complications aiming to increase knowledge about diabetes among foreign‐born persons and thereby improve self‐care behaviour and prevent diabetes‐related complications to improve or retain health. Thus, appropriate diabetes education models, for foreign‐born persons, should be developed and implemented in health care.

## CONFLICT OF INTERESTS

The authors declare that they have no conflict of interest.

## **AUTHOR’S CONTRIBUTION**

SP, EH, HM and KH: Conception and design. SP: Acquisition and collection of data SP, analysis, and interpretation of data. SP, EH, HM and KH: Drafting the article and revising it critically for important intellectual content. All authors have agreed on the final version.
